# Supplementary material for: Cingulate-motor circuits update rule representations for sequential choice decisions
Source: Nat Commun. 2022 Aug 4;13:4545. doi: 10.1038/s41467-022-32142-1 (PMC9352796; doi:10.1038/s41467-022-32142-1)
Supplement: Supplementary file 1 — Supplementary Information [file 41467_2022_32142_MOESM1_ESM.pdf]

Supplementary information for:

Cingulate-motor circuits update rule representations for sequential choice decisions

Daigo Takeuchi, Dheeraj Roy, Shruti Muralidhar, Takashi Kawai, Andrea Bari, Chanel Lovett, Heather A. Sullivan, Ian R. Wickersham & Susumu Tonegawa

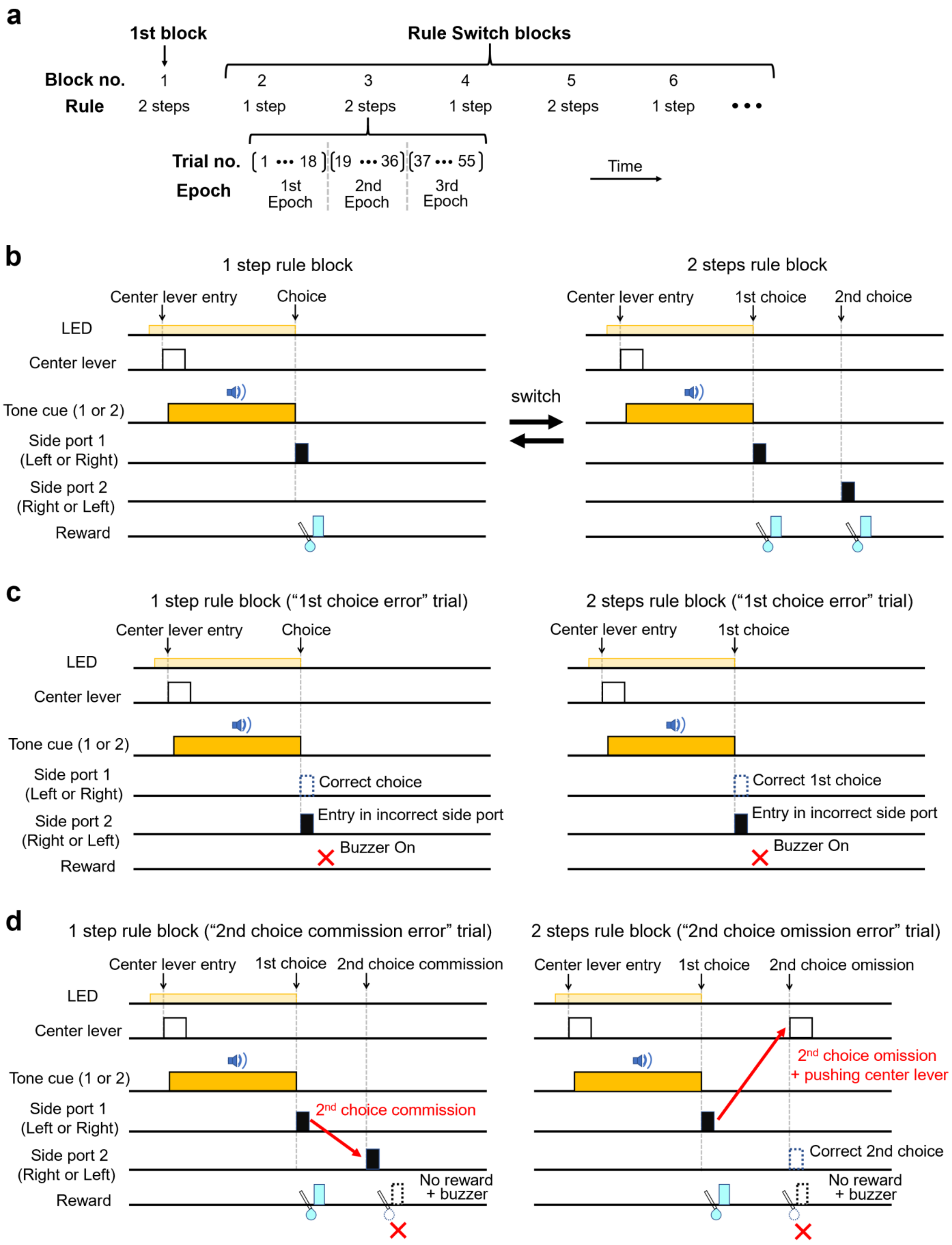
a
1st block
Rule Switch blocks
Block no.
1
2
3
4
5
6
Rule
2 steps
1 step
2 steps
1 step
2 steps
1 step
Trial no.
1 ••• 18
19 ••• 36
37 ••• 55
Epoch
1st Epoch
2nd Epoch
3rd Epoch
Time
b
1 step rule block
Center lever entry
Choice
LED
Center lever
Tone cue (1 or 2)
Side port 1 (Left or Right)
Side port 2 (Right or Left)
Reward
switch
2 steps rule block
Center lever entry
1st choice
2nd choice
c
1 step rule block ("1st choice error" trial)
Center lever entry
Choice
Correct choice
Entry in incorrect side port
Buzzer On
2 steps rule block ("1st choice error" trial)
Center lever entry
1st choice
Correct 1st choice
Entry in incorrect side port
Buzzer On
d
1 step rule block ("2nd choice commission error" trial)
Center lever entry
1st choice
2nd choice commission
2nd choice commission
No reward + buzzer
2 steps rule block ("2nd choice omission error" trial)
Center lever entry
1st choice
2nd choice omission
2nd choice omission + pushing center lever
Correct 2nd choice
No reward + buzzer

Supplementary Fig. 1. Task structure. a, Task rule was switched block-wise between 1 step and 2 steps conditions in every 55 trials. All the blocks succeeding 1st block were classified as Rule Switch-blocks. A block was divided in three epochs (1-18th trial, 19-36th trial and 37-55th trial for 1st, 2nd and 3rd epochs, respectively). **b,** Trials in 1 step (left) and 2 steps (right) blocks. LED onset signals the end of inter trial interval (ITI). A tone cue stimulus (8 or 12 kHz) was presented when rats triggered the center lever. In 1 step condition, rats were required to choose left or right port depending on cue stimulus. If they poked a correct port, a water reward was delivered (correct choice) followed by an ITI. In 2 steps condition, like 1 step condition, rats were required to first choose left or right port depending on cue stimulus and, if they poked a correct one, a water reward is delivered (correct 1st choice). Rats additionally received a 2nd reward if they poked the opposite side port as their 2nd choice. Note that the mapping of cue stimuli to 1st choice responses was unaltered between 1 step and 2 steps conditions; it was the number of response steps that changed between blocks. **c,** 1st choice error in 1 step (left) and 2 steps (right) conditions. In both 1 step and steps conditions, if rats poked an incorrect port as their 1st choice, no water reward but instead a buzzer sound was delivered with an elongated ITI. **d,** Left, 2nd choice commission error in 1 step condition. In 1 step condition, no 2nd reward but instead a buzzer sound was delivered when rats poked the opposite port after making a correct choice and receiving the 1st reward. Right, 2nd choice omission error in 2 steps condition. In 2 steps condition, no reward but instead a buzzer sound was delivered when rats could successfully make a correct 1st choice but failed to make a correct 2nd choice by incorrectly pushing the center lever before poking the opposite port.

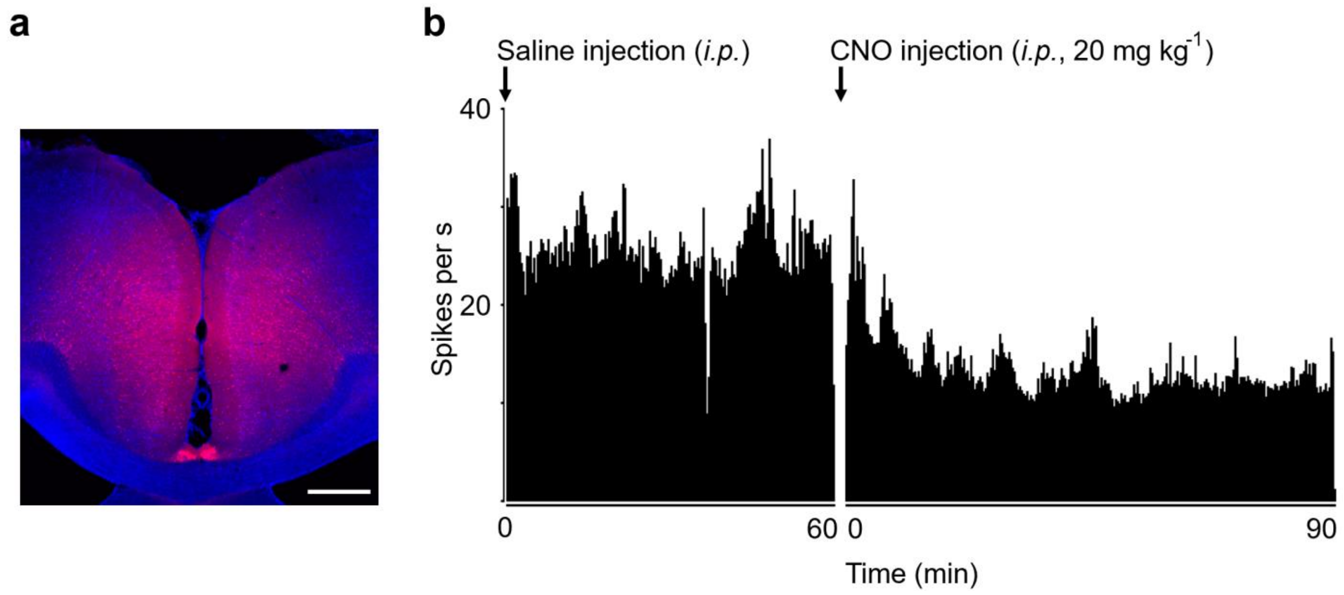

Supplementary Fig. 2. Intraperitoneal injection of clozapine-N-oxide solution suppressed spiking activities in anterior cingulate cortex expressing inhibitory DREADD virus. a, Inhibitory DREADD virus (AAV5-CaMKIIa-hM4Di-mCherry) was bilaterally injected in anterior cingulate cortex (ACC). Red, hM4Di-mCherry expression. Blue, DAPI. Scale bar, 500 μm. **b,** Time histogram of multiunit firing rate measured in ACC expressing inhibitory DREADD virus. First, multiunit spiking activities were measured for 60 minutes after an intraperitoneal (IP) injection of saline solution. Then, clozapine-N-oxide (CNO) solution was injected (IP, 20 mg/kg) and multiunit activity measurement was resumed that lasted for another 90 min. Spiking activity decreased after CNO injection and this effect lasted for at least 60 min after having reached a plateau level (~30 min after an administration of CNO). Bin width, 20 sec. Source data are provided as a Source Data file.

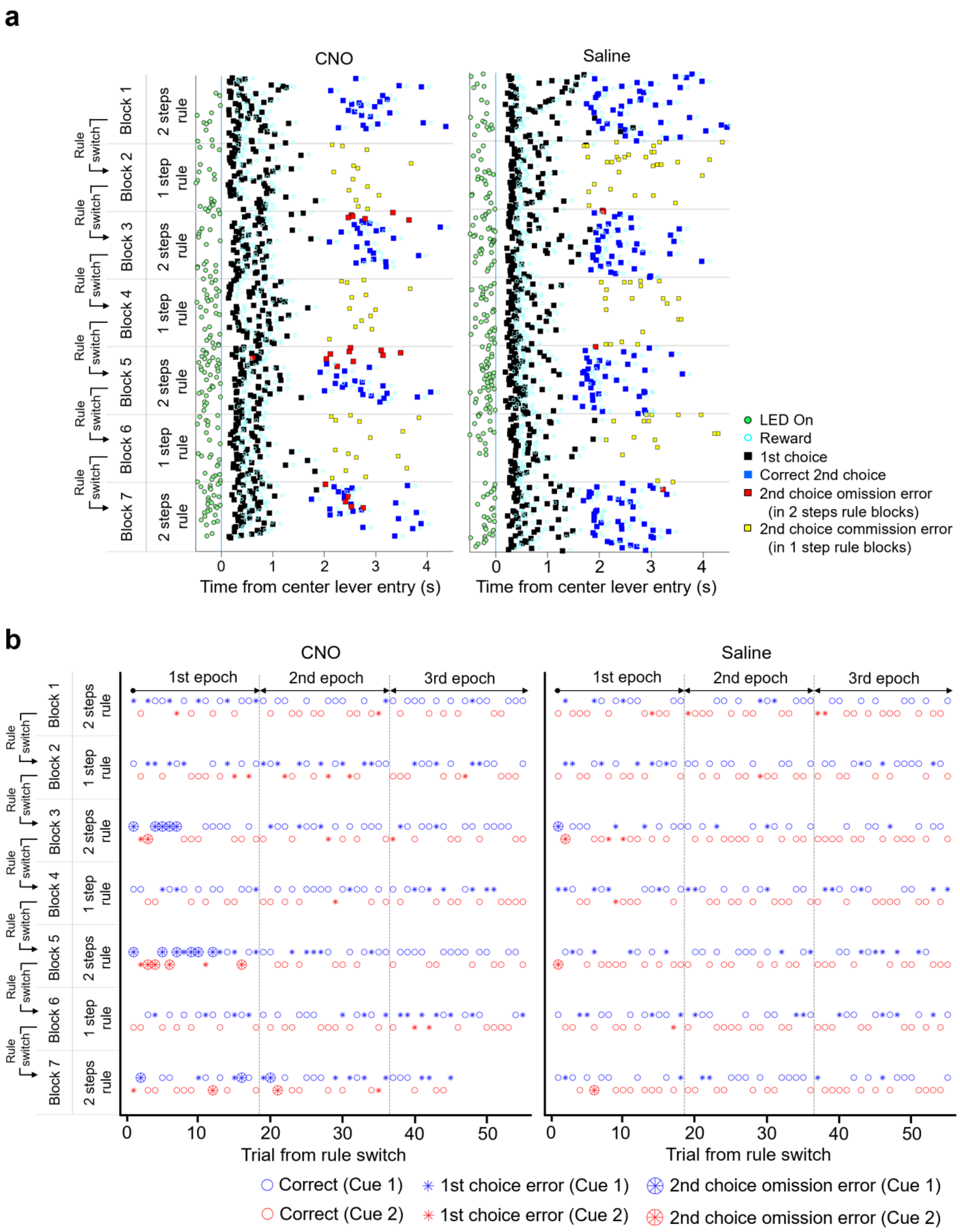
a
CNO
Saline
Block 1
2 steps rule
Block 2
1 step rule
Block 3
2 steps rule
Block 4
1 step rule
Block 5
2 steps rule
Block 6
1 step rule
Block 7
2 steps rule
Rule switch
Time from center lever entry (s)
LED On
Reward
1st choice
Correct 2nd choice
2nd choice omission error (in 2 steps rule blocks)
2nd choice commission error (in 1 step rule blocks)
b
1st epoch
2nd epoch
3rd epoch
Trial from rule switch
Correct (Cue 1)
1st choice error (Cue 1)
2nd choice omission error (Cue 1)
Correct (Cue 2)
1st choice error (Cue 2)
2nd choice omission error (Cue 2)

Supplementary Fig. 3. Task performance charts of representative sessions with chemogenetic silencing of ACC. a, Events in individual trials in two representative sessions (same sessions as presented in Fig. 2b,c) with an IP injection of clozapine-N-oxide (CNO) solution (left, 10 mg/kg) or of a saline solution (right). Task events were sorted by the timing of the animal's pushing the center lever for initiating a trial. Green filled circle, LED onset. Black filled square, 1st choice response. Blue filled square, a correct 2nd choice response. Cyan circle, a water reward. Red filled square, incorrectly pushing the center lever before poking the opposite side port (2nd choice omission error). Yellow filled square, an entry to the opposite side port after making a correct 1st choice in 1 step condition for which no reward was delivered and instead a buzzer sound was presented (2nd choice commission error). Trials were plotted from top to bottom (i.e., the first trial in a session was plotted at the top row). In every 55 trials, task rules were switched between 1 step and 2 steps conditions. **b,** Task performance chart of the same two representative sessions as in **a**. Small circle, correct trial. Asterisk, incorrect 1st choice (1st choice error). Large circle filler with an asterisk, incorrect 2nd choice trial (2nd choice omission error). Blue and red represent trials with two distinct tone cue stimuli. Gray dotted lines distinguish three epochs in each block (1-18th, 19-36th and 37-55th trials for 1st, 2nd and 3rd epoch, respectively).

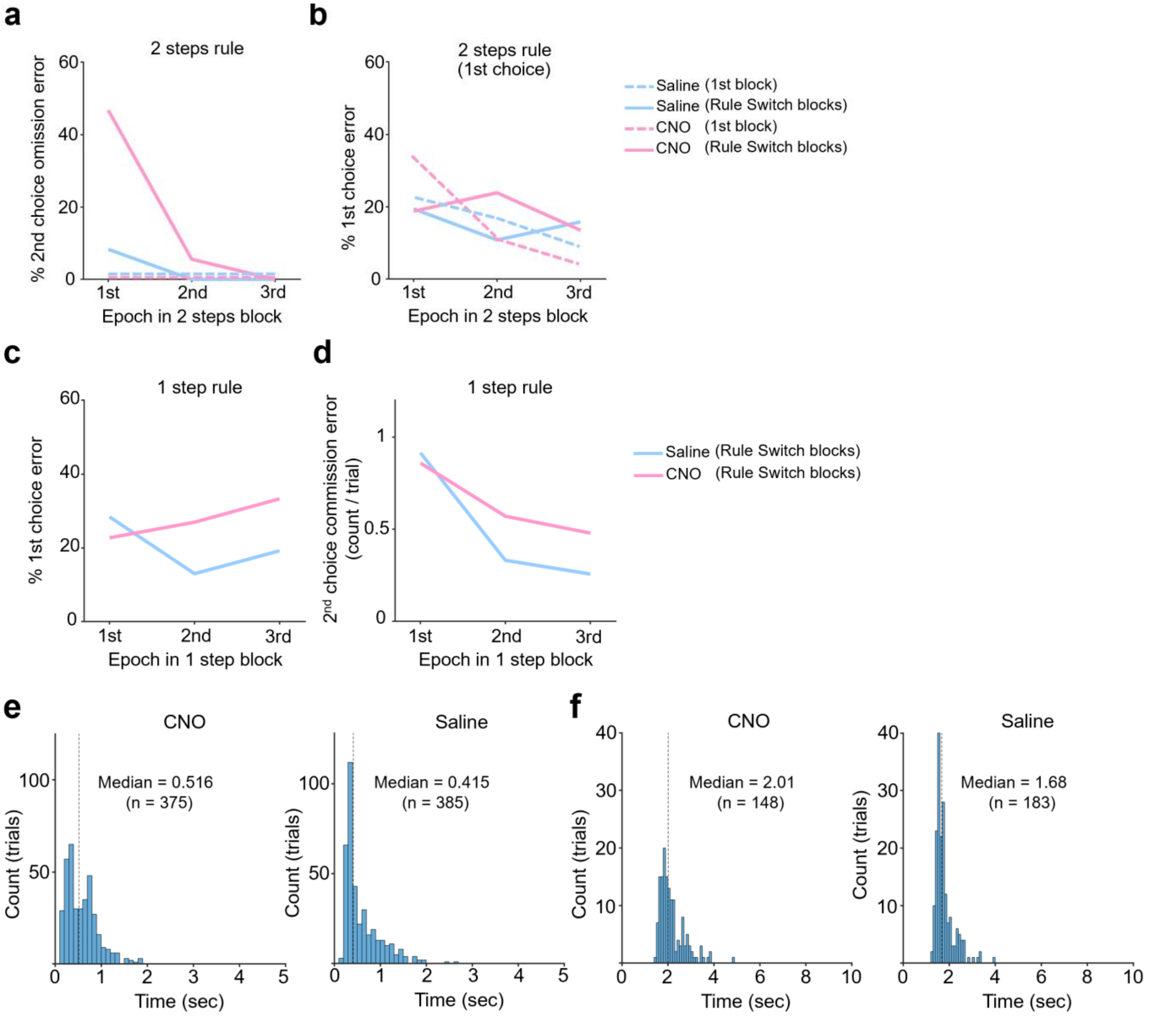

Supplementary Fig. 4. Behavioral effects of chemogenetic silencing of ACC in representative sessions. a, 2nd choice performance in 2 steps condition (%2nd choice omission error) was plotted for two representative sessions (same sessions as presented in Fig. 2b,c and Supplementary Fig. 3) with an IP injection of CNO solution (10 mg/kg, pink) or of a saline solution (blue). Trials in 1st block (dotted red and blue lines) and Rule Switch-blocks (solid red and blue lines) were grouped into three epochs according to the trial no. in the corresponding blocks. 1st, 2nd and 3rd epochs correspond to 1-18th, 19-36th and 37-55th trials, respectively. Percent error rates for two tone cue conditions were averaged. See Fig. 2b for performance in 1st epoch (the first 18 trials). **b**, Same format as in **a**, but average 1st choice performance in 2 steps condition was plotted. **c**, Same format as in **a** and **b**, but average choice performance in 1 step condition was plotted. **d**, Average number of 2nd choice commission errors per trial in 1 step condition was plotted for each of three epochs. See Fig. 2c for performance in 1st epoch (i.e., the first 18 trials in the block). **e**, Histograms of response time for 1st choice. All trials from 1 step and 2 steps conditions were combined. Bin width, 100 ms. **f,** Same format as in **e**, but for 2nd choices of correct trials in 2 steps condition. Source data are provided as a Source Data file.

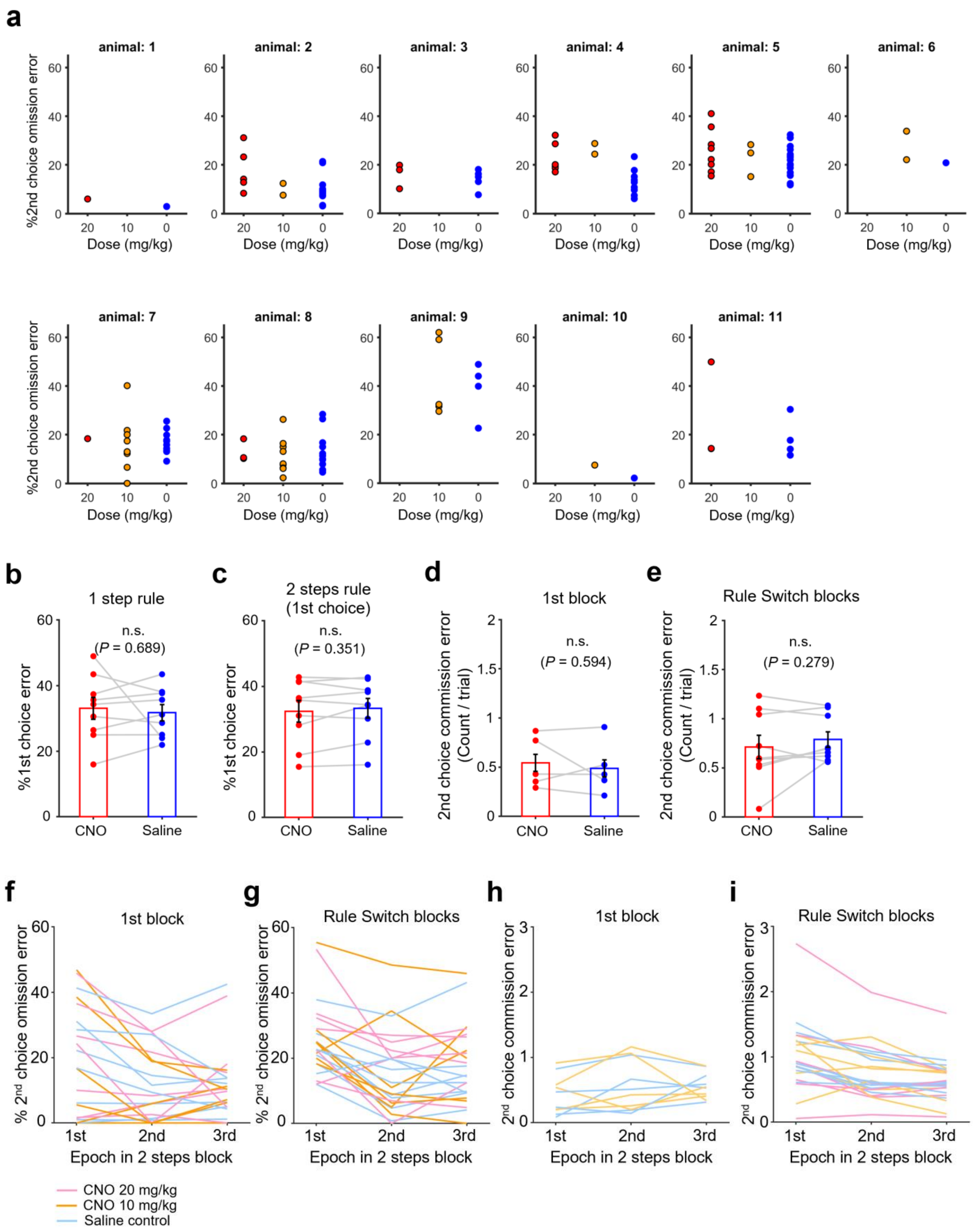
a
animal: 1
animal: 2
animal: 3
animal: 4
animal: 5
animal: 6
animal: 7
animal: 8
animal: 9
animal: 10
animal: 11
%2nd choice omission error
Dose (mg/kg)
b
1 step rule
n.s.
(P = 0.689)
%1st choice error
c
2 steps rule
(1st choice)
n.s.
(P = 0.351)
d
1st block
n.s.
(P = 0.594)
2nd choice commission error
(Count / trial)
e
Rule Switch blocks
n.s.
(P = 0.279)
CNO
Saline
f
1st block
g
Rule Switch blocks
% 2nd choice omission error
h
1st block
i
Rule Switch blocks
2nd choice commission error
Epoch in 2 steps block
1st
2nd
3rd
CNO 20 mg/kg
CNO 10 mg/kg
Saline control

Supplementary Fig. 5. The effect of chemogenetic silencing of ACC neurons on task performance. **a,** 2nd choice performance in 2 steps condition for individual animals. Only animals that were tested with CNO in at least two sessions were included in the group analysis (thus animal no.1 and animal no.10 in **a** were not included). **b,** Task performance in 1 step condition with an IP injection of saline or CNO solutions. For CNO data, sessions with doses of 10 mg/kg and 20 mg/kg were combined. Paired *t*-test (two-sided), n = 9 rats. **c**, Same as in **b**, but 1st choice performance in 2 steps condition was plotted. Paired *t*-test (two-sided), n = 9 rats. **d**, 2nd choice commission error in 1st block of 1 step condition. For CNO data, sessions with doses of 10 mg/kg and 20 mg/kg were combined. Paired *t*-test (two-sided), n = 9 rats. **e,** Same as in **d**, but for Rule Switch-blocks. Paired *t*-test (two-sided), n = 9 rats. **f**, 2nd choice performance for three epochs of 1st block in 2 steps condition. Pink, orange and blue correspond to CNO (20 mg/kg), CNO (10 mg/kg) and saline (n = 7, 6 and 8 rats, respectively). 1st, 2nd and 3rd epochs correspond to 1-18th, 19-36th and 37-55th trials. **g**, Same format as in **e**, but for Rule Switch-blocks. Pink, orange and blue lines represent %2nd choice error rate for individual animals in CNO (20 mg/kg), CNO (10 mg/kg) and saline conditions, respectively (n = 7, 7 and 9 rats, respectively). **h**, 2nd choice commission error for three epochs of 1st block in 1 step condition. Orange and blue lines represent 2nd choice commission error for individual animals in CNO and saline conditions, respectively (n = 5 rats). **i**, Same format as in **h**, but for Rule Switch-blocks. Pink, orange and blue lines represent 2nd choice commission error for individual animals in CNO (20 mg/kg), CNO (10 mg/kg) and saline conditions (n = 7, 7 and 9 rats, respectively). Error bars, SEM (**b**-**e**). Source data are provided as a Source Data file.

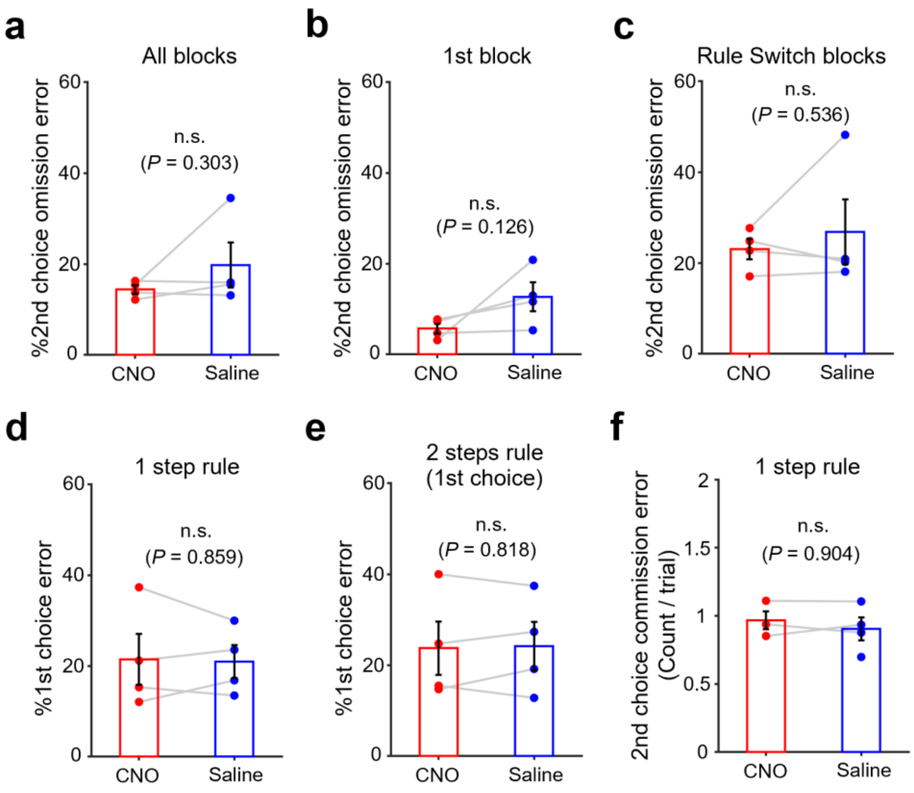

Supplementary Fig. 6. Administration of CNO showed no effect in choice performance in rats injected with control virus in ACC. a, Group result of 2nd choice performance in 2 steps condition (%2nd choice omission error) with an IP injection of saline or CNO solutions in rats injected with AAV5-CamKIIa-mCherry virus in ACC. Percent error rates for two tone cue conditions were averaged. **b,** %2nd choice omission error for trials of 1st block (i.e., non-Rule Switch block) in 2 steps condition. **c,** Same as in **b**, but %2nd choice omission error in Rule Switch-blocks was plotted. **d,** Choice performance in 1 step condition (trials in 1st and Rule Switch-blocks were merged)**. e,** 1st choice performance (%1st choice error) in 2 steps condition (trials in 1st and Rule Switch-blocks were merged). **f,** Group result of average number of 2nd choice commission error per trial in 1 step condition. Paired *t*-test (two-sided), n = 4 rats (**a**-**e**), n = 3 rats (**f**). Error bars, SEM (**a**-**f**). Source data are provided as a Source Data file.

a

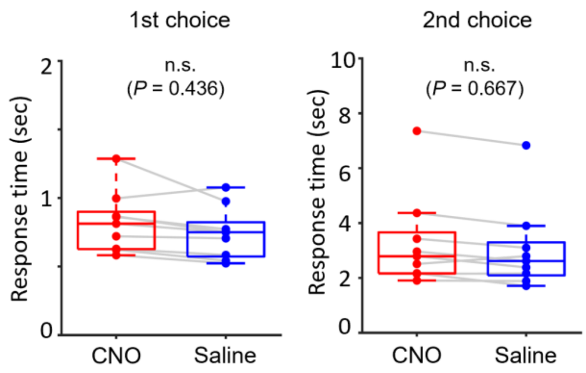

Supplementary Fig. 7. Group results of response time data. a, Group result of response time for 1st choice responses (left) and for 2nd choice responses (right). All the correct trials in 1 step and 2 steps conditions were combined for 1st choice responses. Box-and-whisker plots indicate the minimum, 25th, 50th, 75th percentiles, and maximum. Neither 1st choices nor 2nd choices showed a difference in their response latency between CNO and saline conditions (n = 9 rats, Mann-Whitney's test, two-sided). Source data are provided as a Source Data file.

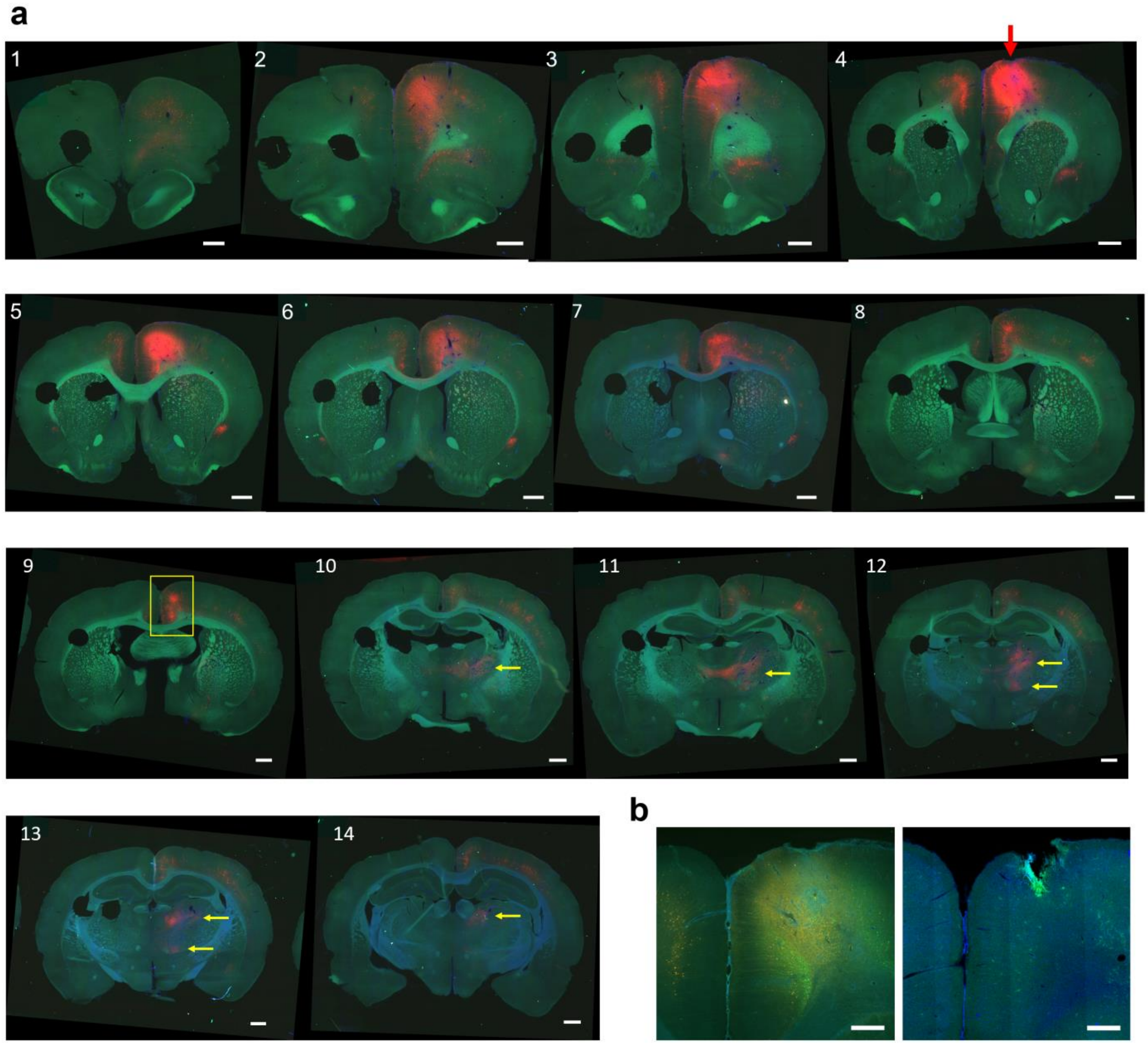

Supplementary Fig. 8. Cingulate and thalamic projections to secondary motor cortex. a, Coronal section series of a rat's brain. A cocktail solution (1 µl) of helper virus (AAV1-synP-FLEX-sTpEpB) and cre virus (pENN.AAV.CaMKII.0.4.Cre.SV40) was injected at secondary motor cortex (M2) followed by injection of genetically modified rabies virus (RV4-mChery (EnvA), 1 µl) at the same coordinate (pointed by a red arrow in panel no. 4). Neurons in ACC (area 24a'/24b') were retrogradely infected with rabies virus expressing mCherry (indicated by a yellow square in panel 9, being also presented in Fig. 3a). Neurons in thalamic nuclei were also retrogradely labelled (pointed by yellow arrows in panels 10-14). GFP (helper virus) and mCherry (Rabies virus) expressions are shown in green and red, respectively. Circular holes on the left side of sections were made before sectioning to identify hemisphere contralateral to virus injections. Scale bar, 1 mm. **b**, Left, magnified view of the virus injection site in M2 (pointed by red arrow in **a**, panel no. 4) (identical image as in Fig. 3b). Right, neither GFP nor mCherry expression was observed near virus injection sites in another wildtype rat in

which helper virus was injected without AAV-CaMKIIa-cre virus. Color codes are the same as in **a**. Each experiment with or without an injection of AAV-CaMKIIa-cre virus was repeated independently using two animals with similar results. Scale bar, 500 μm.

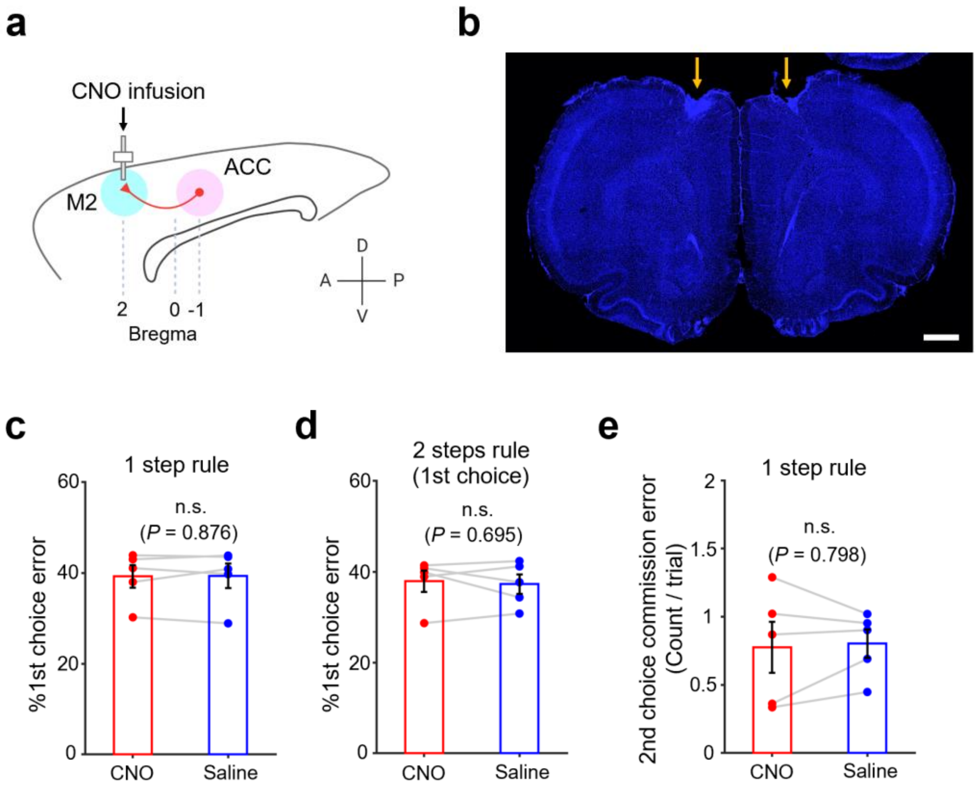

Supplementary Fig. 9. Chemogenetic silencing of ACC neuronal terminals in M2 showed no effect in 1st choice performance and 2nd choice commission error. a, Rats were injected with AAV5-CaMKIIa-hM4Di-mCherry virus in ACC and were implanted with bilateral cannulae in M2. Animals' task performance was tested with a local infusion of either saline or CNO solution (1 µg/µl) in M2. **b,** A coronal section showing tracks (orange arrows) of bilateral cannulae implant in M2. Blue, DAPI. Scale bar, 1 mm. **c**, Group result of choice performance in 1 step condition with local injection of saline or CNO solution in M2. **d**, Group result of 1st choice performance in 2 steps condition. **e,** Group result of average number of 2nd choice commission error per trial in 1 step condition. Paired *t*-test (two-sided), *n* = 5 rats (**c**-**e**). Error bars, SEM (**c**-**e**). Source data are provided as a Source Data file.

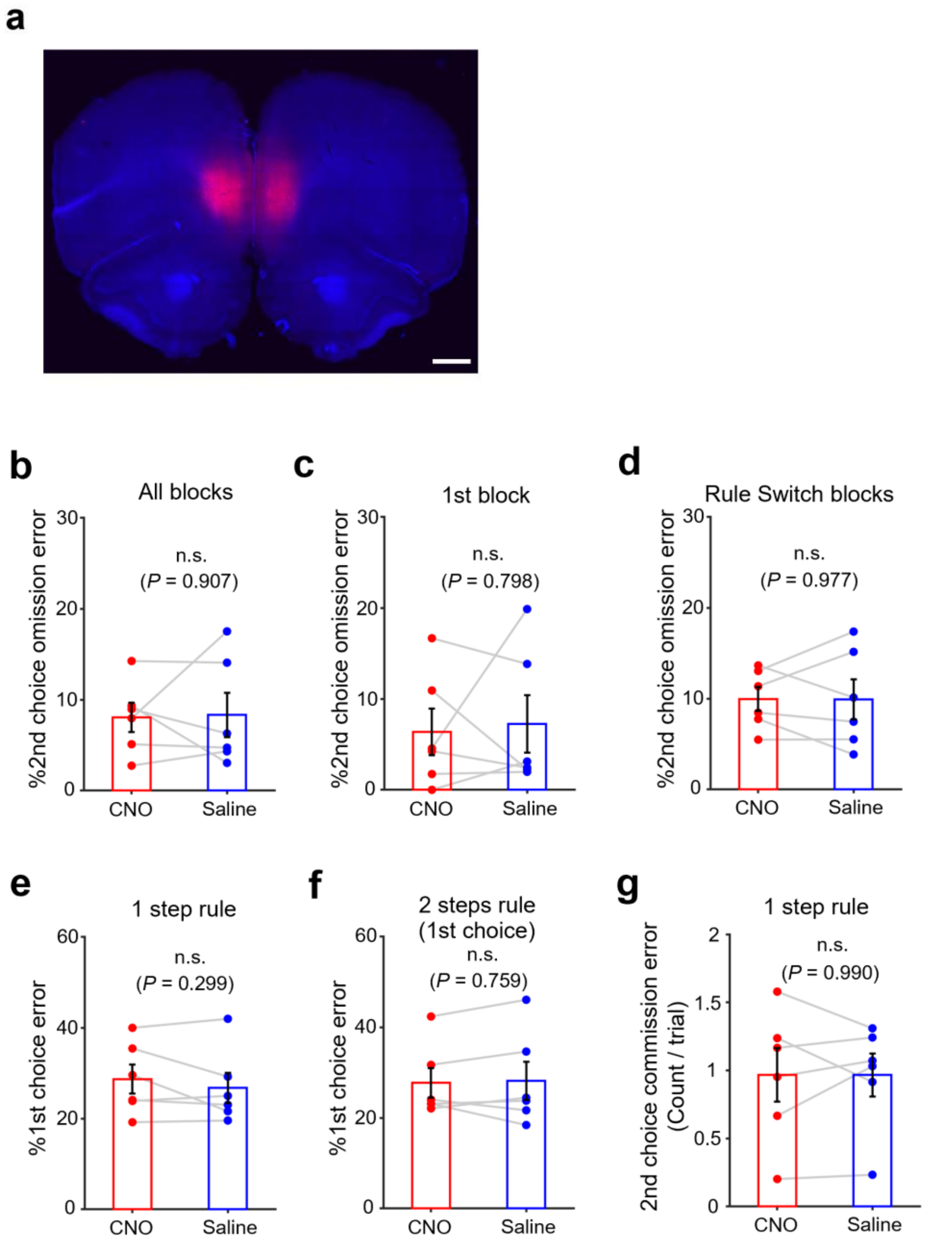

Supplementary Fig. 10. Chemogenetic suppression of prelimbic/infralimbic cortex showed no effect in choice performance. a, AAV5-CaMKIIa-hM4Di-mCherry virus was bilaterally injected in prelimbic/infralimbic cortex (1000 nl for each hemisphere). Red, hM4Di-mCherry expression. Blue, DAPI. Scale bar, 1 mm. **b,** Group result of 2nd choice performance in 2 steps condition (%2nd choice omission error). Percent error rates for two tone cue conditions were averaged. **c,** %2nd choice omission error for trials of 1st block (i.e., non-Rule Switch block) in 2 steps condition was plotted. **d,** Same as in **c**, but %2nd choice omission error in Rule Switch-blocks. **e,** Group result of choice performance in 1 step condition (trials in 1st and Rule Switch-blocks were merged)**. f,** Group result of 1st choice performance (%1st choice error) in 2 steps condition (trials in 1st and Rule Switch-blocks were merged). **g,** Group result of average number of 2nd choice commission error per trial in 1 step condition. Paired *t*-test (two-sided), n = 6 rats (**b-g**). Error bars, SEM (**b-g**). Source data are provided as a Source Data file.

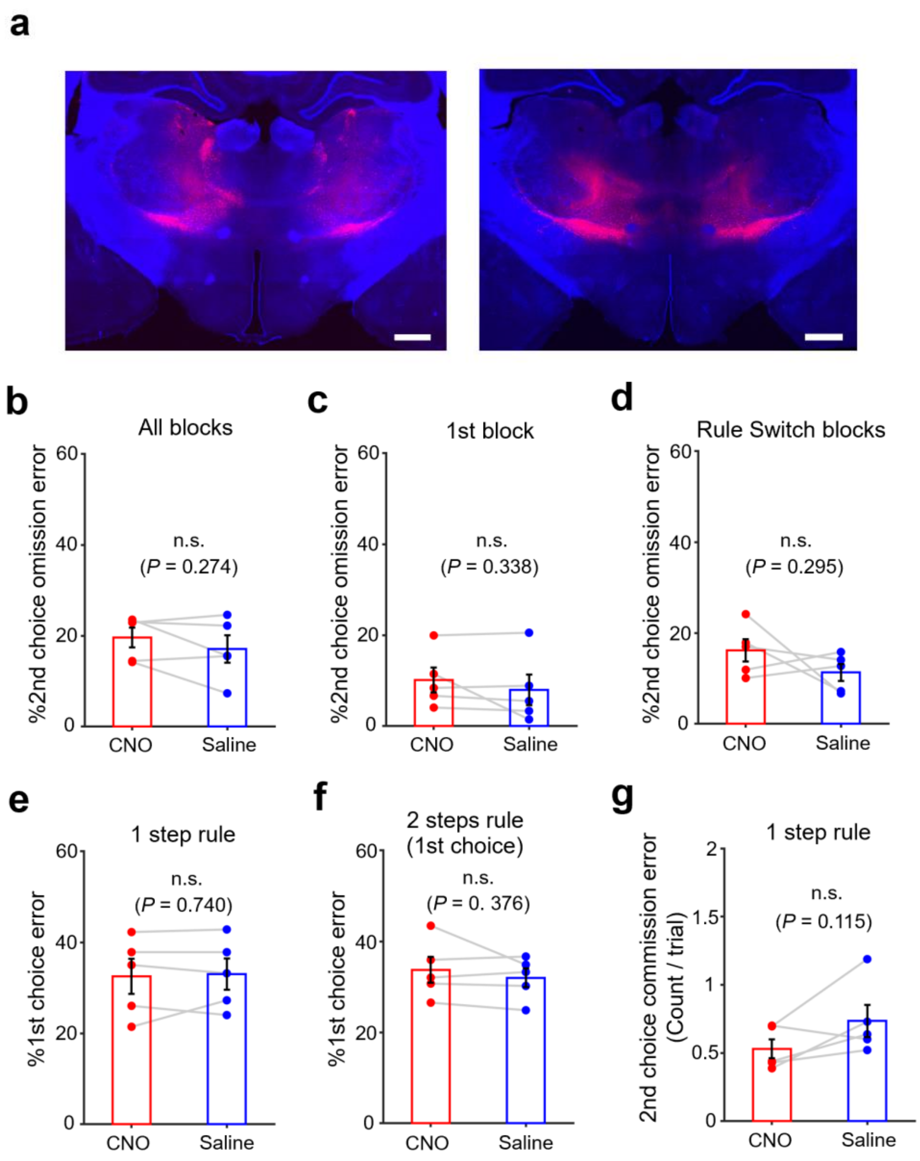

Supplementary Fig. 11. Chemogenetic suppression of ventral thalamus showed no effect in choice performance. a, AAV5-CaMKIIa-hM4Di-mCherry virus was bilaterally injected in ventral thalamic nuclei (1000 nl for each hemisphere). Red, hM4Di-mCherry expression. Blue, DAPI. Scale bar, 1 mm. **b,** Group result of 2nd choice performance in 2 steps condition (%2nd choice omission error) with an IP injection of saline or CNO solutions. Percent error rates for two tone cue conditions were averaged. **c,** %2nd choice omission error for trials of 1st block (i.e., non-Rule Switch block) in 2 steps condition. **d,** Same as in **c**, but %2nd choice omission error in Rule Switch-blocks was plotted. **e,** Group result of choice performance in 1 step condition (trials in 1st and Rule Switch-blocks were merged)**. f,** Group result of 1st choice performance (%1st choice error) in 2 steps condition (trials in 1st and Rule Switch-blocks were merged). **g,** Group result of average number of 2nd choice commission error per trial in 1 step condition. Paired *t*-test (two-sided), n = 5 rats (**b-g)**. Error bars, SEM. Source data are provided as a Source Data file.

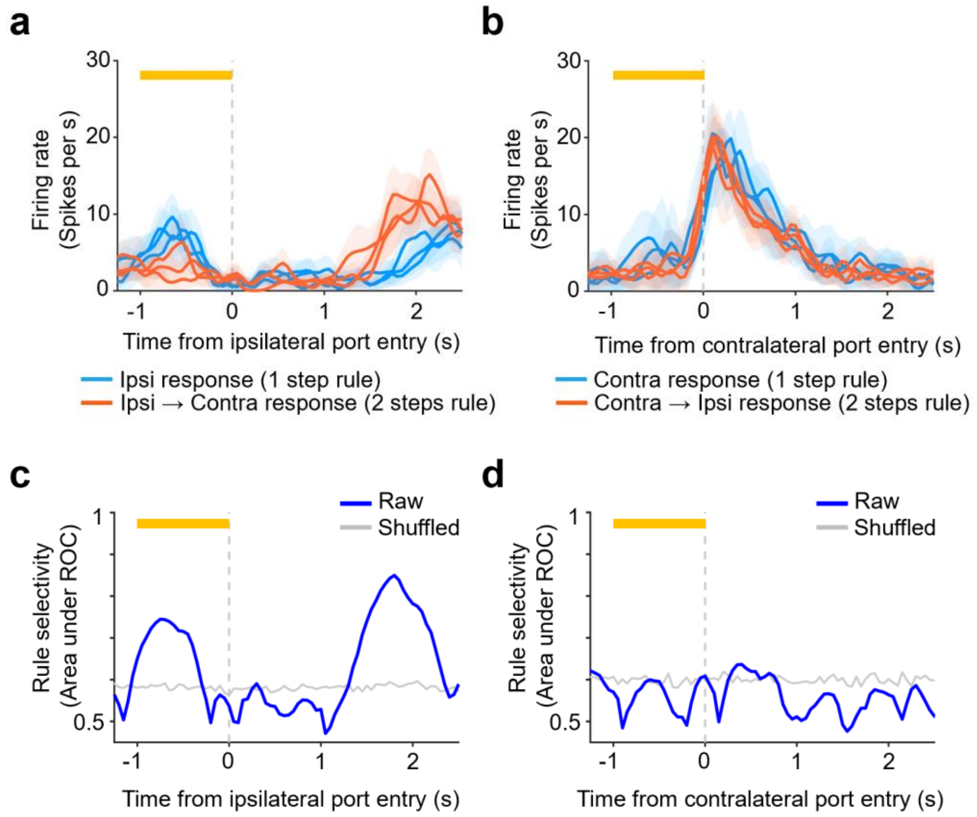

Supplementary Fig. 12. Rule selective activity of example M2 neuron preferring 1 step condition during pre-choice period. a, Peri-event time histogram (PETH) of a representative single-unit showing rule selective responses preferring 1 step condition. PETH was calculated using trials in which the rat made a correct choice of ipsilateral side port, i.e., the side port that was located on the ipsilateral side of neural activity measurements. In 1 step rule block, the rat made a choice to the ipsilateral side port (blue line) while, in 2 steps rule block, the rat made a 1st choice to the ipsilateral side port and then made a 2nd choice to the contralateral side port (red line). Neural activity measured in Rule Switch-block trials were plotted (note that neural activity in 1st block of the session was not included). Each line represents trial-averaged firing rates in a block (three blue lines and three red lines represent three Rule Switch-blocks in 1 step condition and three Rule Switch-blocks in 2 steps condition, respectively). Orange bar at the top indicates pre-choice period, i.e., 1 sec period preceding an animal's entry to the ipsilateral port. Shaded bands, 95% confidence intervals. **b,** Same as in a, but for trials in which the animal made its correct choice response (1 step condition) or correct 1st choice response (2 steps condition) to contralateral side of neural measurements. **c,** Time course of rule selectivity of the single-unit presented in **a** and **b** was plotted for trials in ipsilateral condition. Gray line represents a 95% percentile level estimated by the shuffled data in which the area under ROC curve was calculated with rule labels for trials (i.e., 1 step or 2 steps conditions) being randomly shuffled. **d,** Same as in **c**, but for trials in contralateral condition. Source data are provided as a Source Data file.

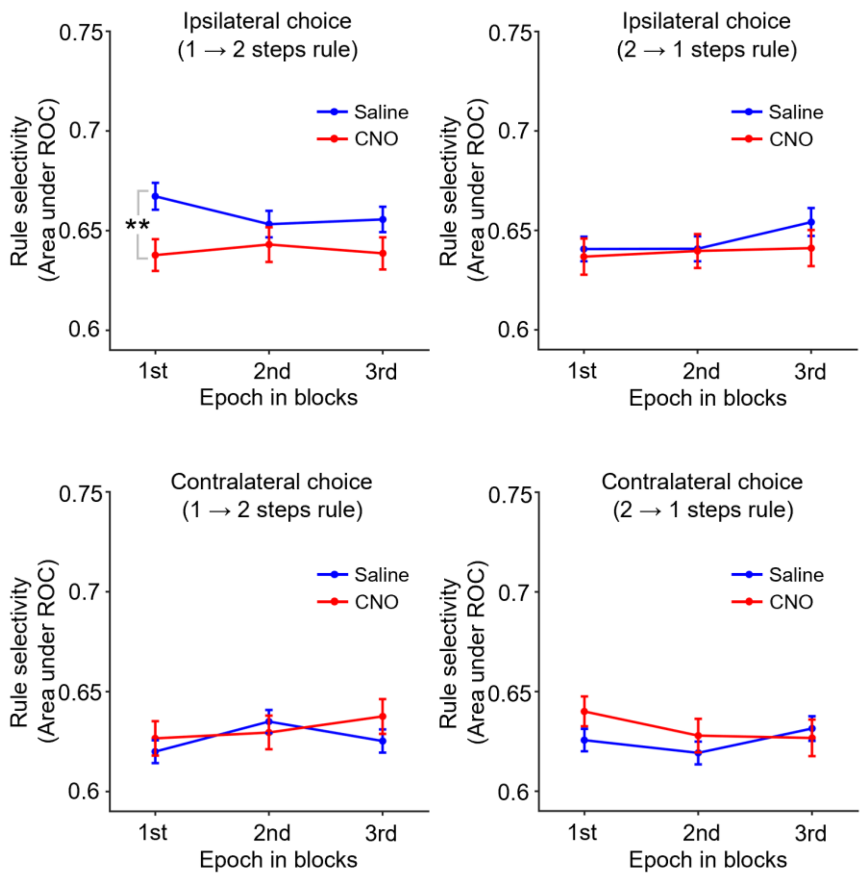

Supplementary Fig. 13. Firing rate-controlled rule selectivity as measured by area under ROC curve. Comparison of rule selectivity between CNO and saline conditions and across three epochs in Rule Switch-blocks. Format is the same as in Fig. 6e, but the mean firing rates across all the trials were adjusted to match between single-units measured in saline and CNO conditions. See Methods for details. A repeated measures two-way ANOVA (with epoch being a within-subject factor) was conducted for ipsilateral condition in blocks following rule switches from 1→2 steps conditions (top left). No interaction was found between CNO dose and epoch in Ipsilateral choice (1→2 steps) condition (P > 0.4). A significant main effect of CNO dose was detected ($F_{1,1833}$ = 7.11, P = 0.0078), but not for epoch (P > 0.2). Post-hoc comparison using two independent samples t-test showed significant differences between saline and CNO solutions in 1st epoch. No significant interaction or main effect was detected for other three conditions (i.e., ipsilateral condition in blocks following rule switches from 2→1 steps condition in top right panel, contralateral condition in blocks following rule switches from 1→2 steps condition in bottom left panel and contralateral condition in blocks following rule switches from 2→1 steps condition in bottom right panel). **P = 0.0083. n = 437 and n = 195 single-units for saline and CNO conditions, respectively. Error bar, SEM. Source data are provided as a Source Data file.

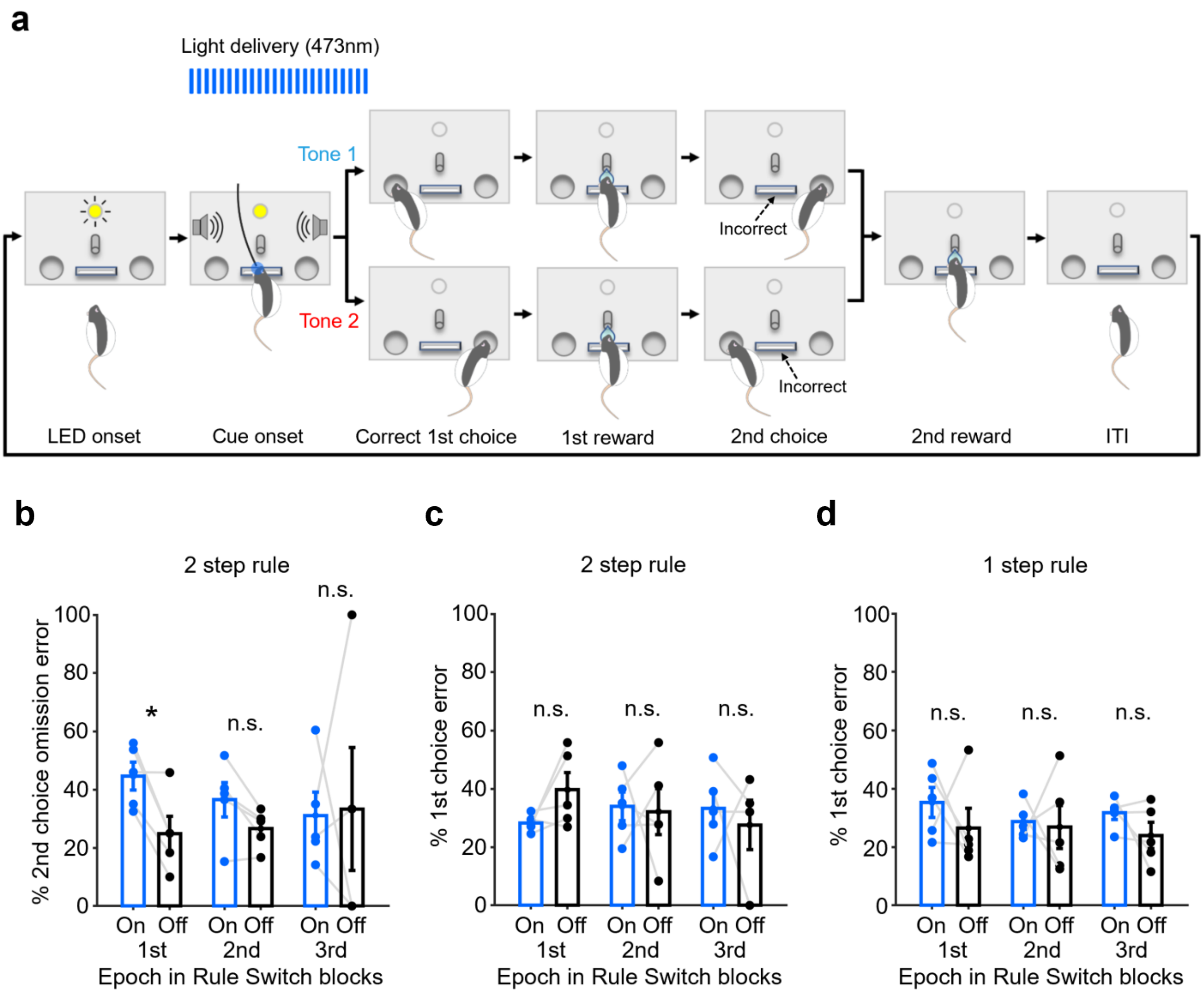

Supplementary Fig. 14. Optogenetic excitation of ACC neuronal terminals in M2 during pre-choice period. a, Optogenetic stimulation of ACC neuronal terminals in M2 during pre-choice period. 473 nm light (10 or 20 Hz, 5 ms pulse) was delivered for 1sec upon cue tone presentation. **b**, %2nd choice omission error was calculated using all the trials with correct 1st choice responses. On and Off represent trial conditions in which light was delivered and not delivered, respectively. Task performance was plotted for each of three epochs separately. *P = 0.031 for 1st epoch, P= 0.171 and 0.925 for 2nd and 3rd epochs, respectively. **c**, Same as in **b**, but for %1st choice error for trials in 2 steps condition. P = 0.094, 0.851 and 0.602 for each epoch, respectively. **d**, Same as in **b**, but for %1st choice error for trials in 1 step condition. P = 0.331, 0.818 and 0.162 for each epoch, respectively. Two samples t-test (two-sided), n = 5 rats (**b**-**d**). Error bars, SEM (**b**-**d**). Source data are provided as a Source Data file.

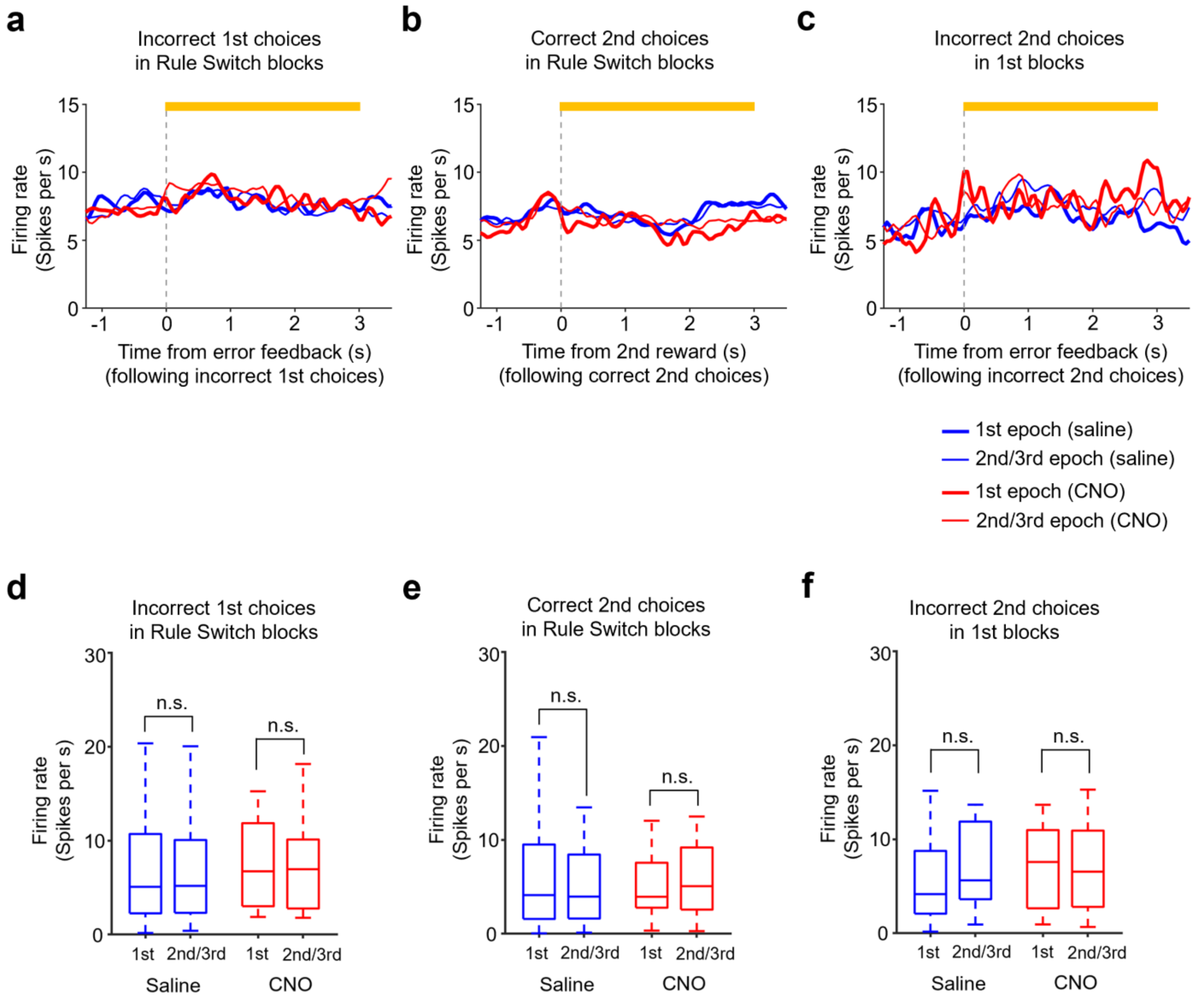

Supplementary Fig. 15. Responses of negative outcome-activated M2 neurons. a, Population-averaged PETHs of negative outcome-activated neurons during negative outcome feedback period following incorrect 1st choices (sorted at error feedback buzzer onset). Blue, saline. Red, CNO. Thick and thin lines, 1st and 2nd/3rd epochs in 2 steps condition, respectively. **b,** Population-averaged PETH of negative outcome-activated neurons during positive outcome feedback period following correct 2nd choices (sorted at 2nd reward onset). **c,** Population-averaged PSTH of negative outcome-activated neurons during negative outcome feedback period following incorrect 2nd choices of 1st block in 2 steps condition. Format is same as in Fig. 7b (bottom left), but for 1st block trials. **d,** Mean firing rate of negative outcome-activated neurons during negative outcome feedback period following incorrect 1st choices of Rule Switch-blocks in 2 steps condition. A significant difference was found neither in saline (median, 5.08 spikes s^{-1} vs 5.18 spikes s^{-1}; n.s., P = 0.66, two-sided Mann-Whitney's test, n = 66 neurons) nor in CNO condition (median, 6.73 spikes s^{-1} vs 6.95 spikes s^{-1}; n.s., P = 0.85, n = 21 neurons). **e,** Mean firing rate of negative outcome-activated neurons during positive outcome feedback

period following correct 2nd choices of Rule Switch-blocks in 2 steps condition. A significant difference was found neither in saline (median, 4.11 spikes s^{-1} vs 3.96 spikes s^{-1}; n.s., $P = 0.90$, two-sided Mann-Whitney's test, $n = 66$ neurons) nor in CNO condition (median, 3.94 spikes s^{-1} vs 5.06 spikes s^{-1}; n.s., $P = 0.23$, $n = 21$ neurons). **f,** Mean firing rate of negative outcome-activated neurons during negative outcome feedback period following incorrect 2nd choices in 1st block. A significant difference was found neither in saline (median, 4.16 spikes s^{-1} vs 5.62 spikes s^{-1}; n.s., $P = 0.99$, two-sided Mann-Whitney's test; $n = 39$ and 31 neurons for 1st and 2/3 epochs, respectively) nor in CNO condition (median, 7.58 spikes s^{-1} vs 6.55 spikes s^{-1}; n.s., $P = 0.84$; $n = 19$ and 20 neurons for 1st and 2nd/3rd epochs, respectively). Box-and-whisker plots indicate the minimum, 25th, 50th, 75th percentiles, and maximum (**d-f**). Source data are provided as a Source Data file.

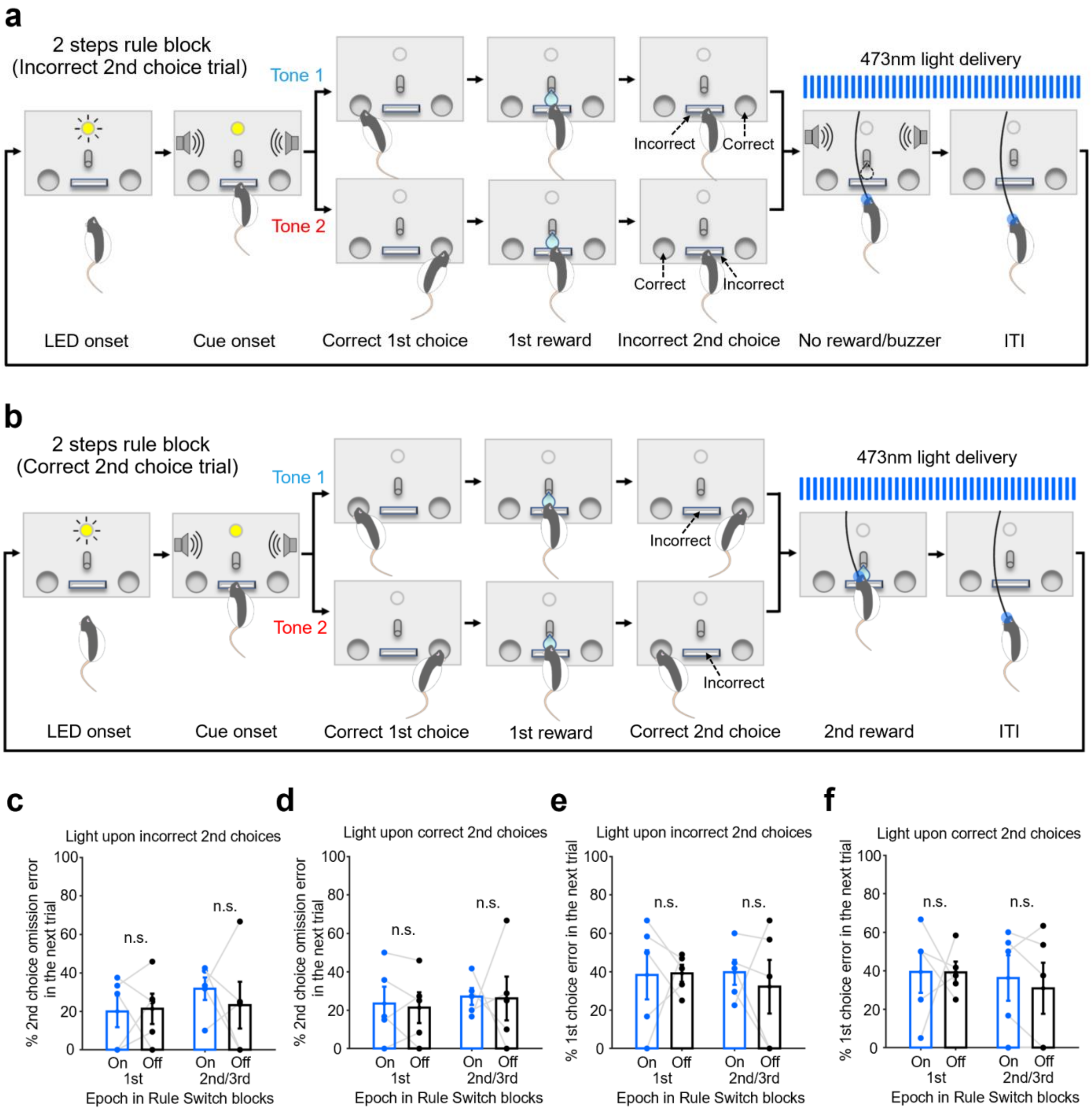

Supplementary Fig. 16. Optogenetic excitation of ACC neuronal terminals in M2 during outcome feedback period following animals' 2nd choices. a, Optogenetic stimulation of ACC neuronal terminals in M2 after animal's incorrect 2nd choices. 473 nm light (10 or 20 Hz, 5 ms pulse) was delivered for 4 sec after incorrect 2nd choices (i.e., animal's pushing the center lever before poking the side port opposite to 1st choice). **b,** Optogenetic stimulation of ACC neuronal terminals in M2 after animal's correct 2nd choices. Light was delivered for 4 sec after correct 2nd choices (i.e., an animal's poking the side port opposite to 1st choice). On and Off represent trial conditions in which light was

delivered and not delivered, respectively. Task performance was plotted for each of three epochs separately. **c**, 2nd choice performance (%2nd choice omission error) was plotted for trials immediately following light-delivered trials (light was delivered upon animals' making a 2nd choice omission error as depicted in **a**). Due to limited number of incorrect 2nd choice trials in 2nd and 3rd epochs, trials in 2nd and 3rd epochs were combined. n.s., P = 0.91 and 0.55 for 1st and 2nd/3rd epochs, respectively. **d**, Same as in **c**, but light was delivered upon animals' making a correct 2nd choice as depicted in **b**. n.s., P = 0.86 and 0.94 for 1st and 2nd/3rd epochs, respectively. **e**, 1st choice performance (% 1st choice error) was plotted for trials immediately following light-delivered trials (light was delivered upon animals' making a 2nd choice omission error as depicted in **a**). n.s., P = 0.95 and 0.64 for 1st and 2nd/3rd epochs, respectively. **f**, Same as in **e**, but light was delivered upon animals' making a correct 2nd choice as depicted in **b**. n.s., P = 0.99 and 0.77 for 1st and 2nd/3rd epochs, respectively. Two samples *t*-test (two-sided), n = 5 rats (**c**-**f**). Error bars, SEM (**c**-**f**). Source data are provided as a Source Data file.
