# Supplementary material for: Cingulate-motor circuits update rule representations for sequential choice decisions
Source: Nat Commun. 2022 Aug 4;13:4545. doi: 10.1038/s41467-022-32142-1 (PMC9352796; doi:10.1038/s41467-022-32142-1)
Supplement: Supplementary file 3 — Description to Additional Supplementary Information [file 41467_2022_32142_MOESM3_ESM.pdf]

Description of Additional Supplementary Files

Source Data Fig.2: data for main Fig. 2

Source Data Fig.4: data for main Fig. 4

Source Data Fig.5: data for main Fig. 5

Source Data Fig.6: data for main Fig. 6

Source Data Fig.7: data for main Fig. 7

Source Data Fig.8: data for main Fig. 8

Source Data Fig.9: data for main Fig. 9

Source Data Fig.S2: data for main Fig. S2

Source Data Fig.S4: data for main Fig. S4

Source Data Fig.S5: data for main Fig. S5

Source Data Fig.S6: data for main Fig. S6

Source Data Fig.S7: data for main Fig. S7

Source Data Fig.S9: data for main Fig. S9

Source Data Fig.S10: data for main Fig. S10

Source Data Fig.S11: data for main Fig. S11

Source Data Fig.S12: data for main Fig. S12

Source Data Fig.S13: data for main Fig. S13

Source Data Fig.S14: data for main Fig. S14

Source Data Fig.S15: data for main Fig. S15

Source Data Fig.S16: data for main Fig. S16
