# Supplementary material for: Cingulate-motor circuits update rule representations for sequential choice decisions
Source: Nat Commun. 2022 Aug 4;13:4545. doi: 10.1038/s41467-022-32142-1 (PMC9352796; doi:10.1038/s41467-022-32142-1)
Supplement: Supplementary file 4 — Reporting Summary [file 41467_2022_32142_MOESM4_ESM.pdf]

nature research

Corresponding author(s): Susumu Tonegawa

Last updated by author(s): Jun 8, 2021

Reporting Summary

Nature Research wishes to improve the reproducibility of the work that we publish. This form provides structure for consistency and transparency in reporting. For further information on Nature Research policies, see our Editorial Policies and the Editorial Policy Checklist.

Statistics

For all statistical analyses, confirm that the following items are present in the figure legend, table legend, main text, or Methods section.

n/a	Confirmed	
☐	☒	The exact sample size (n) for each experimental group/condition, given as a discrete number and unit of measurement
☐	☒	A statement on whether measurements were taken from distinct samples or whether the same sample was measured repeatedly
☐	☒	The statistical test(s) used AND whether they are one- or two-sided<br>*Only common tests should be described solely by name; describe more complex techniques in the Methods section.*
☐	☒	A description of all covariates tested
☐	☒	A description of any assumptions or corrections, such as tests of normality and adjustment for multiple comparisons
☐	☒	A full description of the statistical parameters including central tendency (e.g. means) or other basic estimates (e.g. regression coefficient) AND variation (e.g. standard deviation) or associated estimates of uncertainty (e.g. confidence intervals)
☐	☒	For null hypothesis testing, the test statistic (e.g. F, t, r) with confidence intervals, effect sizes, degrees of freedom and P value noted<br>*Give P values as exact values whenever suitable.*
☒	☐	For Bayesian analysis, information on the choice of priors and Markov chain Monte Carlo settings
☒	☐	For hierarchical and complex designs, identification of the appropriate level for tests and full reporting of outcomes
☒	☐	Estimates of effect sizes (e.g. Cohen's d, Pearson's r), indicating how they were calculated

Our web collection on statistics for biologists contains articles on many of the points above.

Software and code

Policy information about availability of computer code

Data collection	Timing of presentations of sounds from the speakers and delivery of water rewards were controlled using a multifunction digital input/output board with custom programs written in C++ (Visual Studio 2013 Professional, Microsoft) and LabVIEW (LabVIEW 2015 and LabVIEW FPGA Module 2016, National Instruments). The custom behavioral task program codes are available at the following data repository (https://doi.org/10.5281/zenodo.6618578). Neural activity data from task behaving rats were collected using Cheetah 5 software (Neuralynx).
Data analysis	Spike sorting was conducted using Offline Sorter software (ver. 4, Plexon). Spike rastergram (Fig. 8b) and some of the Peri-event time histograms (Fig. 8b and Supplementary Fig. 2b) were created using NeuroExplorer software (ver. 4, Plexon). Intensity of each fluorescence channel in imaging data from immuno-stained brain sections were adjusted using ZEN software (ZEN 2011 Blue edition, Zeiss). ANOVA was conducted using codes written in R (R Studio). All the other analyses were conducted using MATLAB (2013b and 2019a, Mathworks Inc). Data analysis codes (MATLAB and R) are available at https://doi.org/10.17605/OSF.IO/EVC73.

For manuscripts utilizing custom algorithms or software that are central to the research but not yet described in published literature, software must be made available to editors and reviewers. We strongly encourage code deposition in a community repository (e.g. GitHub). See the Nature Research guidelines for submitting code & software for further information.

Data

Policy information about availability of data

All manuscripts must include a data availability statement. This statement should provide the following information, where applicable:

- Accession codes, unique identifiers, or web links for publicly available datasets
- A list of figures that have associated raw data
- A description of any restrictions on data availability

Data used in this study is available at https://doi.org/10.17605/OSF.IO/EVC73.

Field-specific reporting

Please select the one below that is the best fit for your research. If you are not sure, read the appropriate sections before making your selection.

- [x] Life sciences
- [ ] Behavioural & social sciences
- [ ] Ecological, evolutionary & environmental sciences

For a reference copy of the document with all sections, see nature.com/documents/nr-reporting-summary-flat.pdf

Life sciences study design

All studies must disclose on these points even when the disclosure is negative.

Sample size	Sample sizes were selected based on our knowledge of rat-to-rat variability in behavior obtained in pilot experiments for developing the behavior task. This led us to include 31 rats for chemogenetic experiments and 10 rats for optogenetic experiments. Among 31 rats for chemogenetic experiments, 15 rats were injected with inhibitory DREADD virus in anterior cingulate cortex, 5 rats were injected with a control virus in the same site, 6 and 5 rats were injected with inhibitory DREADD virus in prelimbic/infralimbic cortex and ventral thalamus, respectively. Among 15 rats that were injected with inhibitory DREADD virus in anterior cingulate cortex, 10 rats were used for intraperitoneal injection of clozapine-N-oxide (CNO) solution, 4 rats were used for local infusiointran experiments and one rat were used for both experiments. Among 11 rats that were injected with inhibitory DREADD virus in anterior cingulate cortex and tested on intraperitoneal injection of CNO, 5 rats were used for neural activity measurements during task performance. Among 10 rats for optogenetic experiments, 5 rats were injected with halorhodopsin virus (eNpHR3.0) in anterior cingulate cortex and the other 5 rats were injected with channelrhodopsin virus (hChR2) in anterior cingulate cortex.
Data exclusions	All animals tested were included in the study except that two animals injected with with inhibitory DREADD virus in anterior cingulate cortex were excluded from the group analysis due the small number of CNO sessions (the two excluded rats were tested with CNO only in single session). Behavioral sessions with trial number of equal to or fewer trials than 155 were excluded from the group analysis so that we could analyze and compare rats' task performance across several epochs within a behavioral task block. In chemogenetic experiment, two animals were tested with a higher dose of intraperitoneal administration of CNO solution (40 mg/kg). They were excluded from group analysis due to the small sample size. Similarly, one rat was tested with lower (0.4ug/ul and 4ug/ul) doses of local infusion of CNO solution but was excluded from the analysis due to the small sample size. In electrophysiological data analysis, all the isolated singule-units were included for analysis of the effect of CNO administration on firing rate. In the analysis of the effect of CNO administration on rule selectivity, single-units with a baseline firing rate of less than 3 Hz (measured during a 1sec period immediately before animal's making the 1st choice) were excluded. Similarly, in the analysis of the effect of CNO administration on outcome selectivity, single-units with a baseline firing rate of less than 1 Hz (measured during a 1sec period starting from 1.5sec before animal's initiating a trial) were excluded from the analysis.
Replication	No attempt at replication of the exact results has been done outside of our study. However, we are reporting results for all individual subjects, as well as the average effect, in behavioral assessments with and without chemogenetic inactivations, neural activity measurements, optogenetic inactivations and stimulations, and show how consistent are the results across different subjects.
Randomization	Animals were randomly assigned to cohorts in chemogenetic experiments that were injected with inhibitory DREADD virus and its control virus and in optogenetic experiments (optogenetic inactivations and excitations). The hemispheres that were implanted with electrode arrays in electrophysiological experiments were randomly assigned in each rat (two and three rats were implanted with the array electrodes in left and right hemispheres, respectively).
Blinding	Blinding was not performed. However, the behavioral data collection process was entirely computer-controlled and automatic and, by doing so, any potential bias in behavioral assessment and data analysis caused by investigators' knowledge of animals' identity was minimized.

Reporting for specific materials, systems and methods

We require information from authors about some types of materials, experimental systems and methods used in many studies. Here, indicate whether each material, system or method listed is relevant to your study. If you are not sure if a list item applies to your research, read the appropriate section before selecting a response.

Materials & experimental systems

n/a	Involved in the study	
[ ]	[x]	Antibodies
[x]	[ ]	Eukaryotic cell lines
[x]	[ ]	Palaeontology and archaeology
[ ]	[x]	Animals and other organisms
[x]	[ ]	Human research participants
[x]	[ ]	Clinical data
[x]	[ ]	Dual use research of concern

Methods

n/a	Involved in the study	
[x]	[ ]	ChIP-seq
[x]	[ ]	Flow cytometry
[x]	[ ]	MRI-based neuroimaging

Antibodies

Antibodies used	Primary rabbit anti-RFP antibody (Rockland, 600-401-379).<br>(https://rockland-inc.com/store/Antibodies-to-GFP-and-Antibodies-to-RFP-600-401-379-O4L_24299.aspx)<br><br>Primary chicken anti-GFP (Life Technologies, A10262).<br>(https://www.thermofisher.com/antibody/product/GFP-Antibody-Polyclonal/A10262)<br><br>Secondary anti-rabbit Alexa 555 (Life Technologies, A21429).<br>(https://www.thermofisher.com/antibody/product/Goat-anti-Rabbit-IgG-H-L-Highly-Cross-Adsorbed-Secondary-Antibody-Polyclonal/A-21429)<br><br>Secondary anti-rabbit Alexa 568 (Life Technologies, A11011).<br>(https://www.thermofisher.com/antibody/product/Goat-anti-Rabbit-IgG-H-L-Cross-Adsorbed-Secondary-Antibody-Polyclonal/A-11011)<br><br>Secondary anti-chicken Alexa 488 (Life Technologies, A11039).<br>(https://www.thermofisher.com/antibody/product/Goat-anti-Chicken-IgY-H-L-Secondary-Antibody-Polyclonal/A-11039)
Validation	All these antibodies have been validated by the manufacturer and other researchers using cell lines, western blots, and mouse or rat tissues.

Animals and other organisms

Policy information about studies involving animals; ARRIVE guidelines recommended for reporting animal research

Laboratory animals	Male Long Evans rats aged 3-5 months (prior to behavioral training and surgery) were used in this study. Rats were kept on a reverse 12 h light/dark cycle, and trained and tested in their dark cycle. Food was available ad libitum, and rats had scheduled access to water for motivating them to work for water reward while monitoring their body weight to ensure they were over 85% of initial weight.
Wild animals	No wild animals were used in this study.
Field-collected samples	No field-collected samples were used in this study.
Ethics oversight	All experiments were conducted in accordance with U.S. National Institutes of Health (NIH) guidelines and the Massachusetts Institute of Technology Department of Comparative Medicine and Committee of Animal Care (the approved protocol no., Tonegawa 0121-006-24).

Note that full information on the approval of the study protocol must also be provided in the manuscript.
